# Reversible Photo-Switching of Dual-Color Fluorescent Mn-Doped CdS-ZnS Quantum Dots Modulated by Diarylethene Molecules

**DOI:** 10.3389/fchem.2019.00145

**Published:** 2019-03-20

**Authors:** Yucheng Yuan, Hua Zhu, Yasutaka Nagaoka, Rui Tan, Andrew Hunter Davis, Weiwei Zheng, Ou Chen

**Affiliations:** ^1^Department of Chemistry, Brown University, Providence, RI, United States; ^2^Department of Chemistry, Syracuse University, Syracuse, NY, United States

**Keywords:** reversible photo-switching, dual-color, Mn-doped CdS-ZnS quantum dots, diarylethene switches, förster resonance energy transfer (FRET), photon reabsorption

## Abstract

Dynamic materials have been given an increased amount of attention in recent years with an expectation that they may exhibit properties on demand. Especially, the combination of fluorescent quantum dots (QDs) and light-responsive organic switches can generate novel photo-switchable materials for diverse applications. In this work, a highly reversible dynamic hybrid system is established by mixing dual-color emitting Mn-doped CdS-ZnS quantum dots (QDs) with photo-switchable diarylethene molecules. We show that the diarylethene 1,2-bis(5-(3,5-bis(trifluoromethyl)phenyl)-2-methylthiophen-3-yl)cyclopent-1-ene (switch molecule **1**) performs fabulous photo-switching property (between its open, **1o** and closed, **1c** forms), and high fatigue resistance in this hybrid system. The emission color switching between blue and pink of the system can be induced mainly by selective quenching/recovering of the Mn- photoluminescence (PL) of the QDs due to the switchable absorbance of the molecule **1**. Mechanistic studies show that quenching of QD emission following UV illumination was caused by both Förster resonance energy transfer (FRET) and reabsorption by surrounding **1c** molecules in the case of the Mn-PL, and solely by reabsorption in the case of badngap- (BG-)PL. This photo-switchable system could be potentially used in applications ranging from self-erasing paper to super-resolution fluorescence imaging.

## Introduction

Dynamic materials have attracted attention of chemists in the past two decades owing to their potentials to be used for generating responsive materials for various applications (Kay et al., 2007; Grinthal and Aizenberg, 2013; Klajn, 2014). Meanwhile, there is comprehensive development in the syntheses of nanocrystals with controlled size, shape, structure and compositions in various material systems (Gilroy et al., 2016; Pietryga et al., 2016; Talapin and Shevchenko, 2016; Wu et al., 2016). Combining organic molecular switches with inorganic nanocrystals can therefore afford dynamic hybrid systems with switchable properties (Klajn et al., 2010; Qu et al., 2015; Bai et al., 2016; Li et al., 2017). Compared to static counterparts, dynamic materials exhibit intriguing advantages including selectively reversible properties with external stimuli, and thus potential applications in various fields including self-erasing paper (Kundu et al., 2015), self-healing coating (Roy et al., 2015) bio-imaging etc. (Zhang et al., 2012). Among diverse stimuli, light has been favored as an external input to tune the state of materials on account of its non-chemical contaminations, convenient delivery and specificity of desired wavelengths. As a result, a variety of photo-switchable molecules such as azobenzenes (Bandara and Burdette, 2012), spiropyrans (Minkin, 2004), dithienylethenes (Irie, 2000; Irie et al., 2014), stilbene (Momotake and Arai, 2004) etc. have been employed to functionalize different nanomaterials (Yildiz et al., 2009; Klajn et al., 2010; Wang and Li, 2018) to construct light-responsive systems spanning from metal nanocrystals (Kundu et al., 2015; Manna et al., 2015; Zhao et al., 2015), metal oxide nanocrystals (Mikami et al., 2004; Min Yeo et al., 2008), to quantum dots (QDs) (Zhu et al., 2005, 2006; Díaz et al., 2011, 2015) and metal-organic frameworks (MOFs) (Dolgopolova et al., 2018).

In materials science, doping is a process that intentionally introduces impurity atoms as dopants to host lattices, thus providing unique properties inaccessible to conventional materials (Bryan and Gamelin, 2005). In this regard, doping in QDs may exhibit improved optical, magnetic and electronic properties as compared to their undoped counterparts (Yang et al., 2006; Pradhan and Peng, 2007; Bussian et al., 2008; Norris et al., 2008; Zheng and Strouse, 2011; Cai et al., 2018; Kroupa et al., 2018; Li et al., 2018). For example, when introducing transition metals or rare earth elements to the QDs, a new emission band may emerge, resulting a dual-color emission property with two tunable non-overlapping photoluminescence (PL) peaks (Wu and Yan, 2013). To this extent, Mn-doped CdS-ZnS QDs have been extensively studied as a model system for both fundamental understanding of host-to-dopant energy transfer processes and practical applications that are utilizing their dual-color emission properties (Yang et al., 2006, 2008; Chen O. et al., 2010; Chen et al., 2012; Hofman et al., 2017; Pradhan et al., 2017).

Taking advantages of the dynamic switchable absorbance of diarylethene molecules and the dual-color emission property of Mn-doped CdS-ZnS QDs, in this work, we demonstrate a photo-switchable hybrid system by selectively quenching/recovering the Mn dopant emission. The quenching process is shown to include Förster resonance energy transfer (FRET) from Mn-PL to closed isomer of diarylethenes as well as reabsorption processes. Three diarylethene switches with different electron-withdrawing groups were exploited to examine the performances of the photo-switching process. We demonstrate that the switch diarylethene molecule with substituent of 3,5-bis(trifluoromethyl)phenyl (switch **1**) afford the best fatigue-resistant performance. PL color switching between blue and pink can be obtained by sequential illumination of UV (365 nm) and visible (590 nm) light. This switchable dual-color dynamic system could be potentially useful in a broad range of applications.

## Materials and Methods

### Materials

All chemicals were used without further purification. 2-Chloro-5-methylthiophene (> 96.0%) was purchased from TCI Chemicals. Tributyl borate (98%) was purchased from Strem Chemicals. 1-Bromo-3,5-bis(trifluoromethyl)benzene (99%), *n-*butyllithium (1.6 M solution in hexanes, AcroSeal), zinc dust (98+%), and tetrahydrofuran (THF, 99.9%, extra dry, stabilized, anhydrous, SC, AcroSeal) were purchased from Acros Organics. Tetrakis(triphenylphosphine) palladium(0) (Pd(PPh_3_)_4_, 99%), glutaryl chloride (97%), TiCl_4_ (99.9% trace metals basis), sulfur powder (99.999%), 1-octadecence (ODE, tech. 90%), and oleylamine (OAm, tech. 70%) were purchased from Aldrich. Manganese acetate tetrahydrate (99%), sodium carbonate (99.8%), and all the solvents were purchased from Fisher Scientific Company. Ethyl 4-bromobenzoate (98+%), AlCl_3_ (anhydrous, 99.985%), cadmium acetate hydrate (99.999%), cadmium oxide (99.998%), zinc stearate (count as ZnO ≈ 14%), oleic acid (OLA, 90%) and selenium (200 mesh, 99.999%) were purchased from Alfa Aesar. Nitric acid (≥69.5%, TraceSELECT) was purchased from Fluka. 4-bromo-pyridine hydrochloride (98%) was purchased from Matrix Scientific. Cadmium myristate was self-made according to the literature method (Chen et al., 2008).

### Synthesis of Diarylethene Molecules

Diarylethenes were synthesized using a previous established route with minor modifications (see Supplementary Material) (Tam et al., 2011). The reactions were carried out using Schlenk line under dry N_2_ flow. *n-*Butyllithium (1.6 M in hexane, 1.3 mL) was added to a solution of 1,2-bis(5-chloro-2-methyl-3- thienyl)cyclopentene (see Supplementary Material for the synthesis, 1.0 mmol) in THF (15 mL) at room temperature. After stirring for 15 min, tributyl borate (3.0 mmol) was added and followed by stirring for another 1 hr. In another flask, DMSO (25 mL) was added and degassed, then 1-bromo-3,5-bis(trifluoromethyl)benzene (2.2 mmol) and Pd(PPh_3_)_4_ (0.02 mmol) were added. After stirring for 15 min, aqueous Na_2_CO_3_ (2 M, 5 mL) and ethylene glycol (0.5 mL) were added. The mixture was heated up to 60°C after stirring for 15 min, then was added the above prepared solution. The resulting mixture was stirred at 80°C overnight. After cooling to room temperature, 50 mL of water was added and the mixture was extracted with 20 mL of ethyl acetate three times. The combined organic phases were washed with brine, dried over Na_2_SO_4_ and evaporated. Purification by flash column chromatography afforded compound 1,2-bis(5-(3,5-bis(trifluoromethyl)phenyl)-2-methylthiophen-3-yl)cyclopent-1-ene (**1o**) (32%) as a pale white solid (Herder et al., 2015). ^**1**^**H-NMR (400 MHz, CDCl**_**3**_**):** δ (ppm) = 7.86 (br s, 4 H, C*H*_ar_), 7.71 (br s, 2 H, C*H*_ar_), 7.14 (s, 2 H, C*H*_th_), 2.88 (t, *J*_*H, H*_ = 7.4 Hz, 4 H, C*H*_2_), 2.14 (p, *J*_*H, H*_ = 7.2 Hz, 2 H, C*H*_2_), 2.06 (s, 6 H, C*H*_3_). Compound 1,2-bis(2-methyl-5-(pyridin-4-yl)thiophen-3-yl)cyclopent-1-ene (**2o**, in Supplementary Material) was prepared by exchanging 1-bromo-3,5-bis(trifluoromethyl)benzene with 4-bromo-pyridine hydrochloride (Tam et al., 2011). ^1^H-NMR (400 MHz, CDCl_3_): δ (ppm) = 8.53 (d, *J*_*H, H*_ = 6.0 Hz, 4H, C*H*_ar_), 7.39 (d, *J*_*H, H*_ = 6.4 Hz, 4H, C*H*_ar_), 7.25 (s, 2H, C*H*_th_), 2.86 (t, *J*_*H, H*_ = 7.4 Hz, 4H, C*H*_2_), 2.12 (m, 2H, C*H*_2_), 2.03 (s, 6H, C*H*_3_). Compound 4,4′-(cyclopent-1-ene-1,2-diylbis(5-methylthiophene-4,2-diyl))dibenzoic acid (**3o**, in Supplementary Material) was obtained by switching 1-bromo-3,5-bis(trifluoromethyl)benzene to ethyl 4-bromobenzoate to perform the Suzuki reaction and followed by hydrolysis with 4 M NaOH aqueous solution in dioxane (Mulder et al., 2004; Herder et al., 2015). ^**1**^**H-NMR (400 MHz, CDCl**_**3**_**):** δ (ppm) = 7.99 (d, *J*_*H, H*_ = 8.8 Hz, 4 H, C*H*_ar_), 7.53 (d, *J*_*H, H*_ = 8.8 Hz, 4 H, C*H*_ar_), 7.14 (s, 2 H, C*H*_th_), 4.37 (q, *J*_*H, H*_ = 7.2 Hz, 4 H, CH_3_C*H*_2_O), 2.85 (t, *J*_*H, H*_ = 7.4 Hz, 4 H, C*H*_2_), 2.10 (p, *J*_*H, H*_ = 7.4 Hz, 2 H, C*H*_2_), 2.01 (s, 6 H, C*H*_3_), 1.39 (t, *J*_*H, H*_ = 7.2 Hz, 6 H, C*H*_3_CH_2_O). ^**1**^**H NMR (400 MHz, DMSO-*d***^**6**^**):** δ (ppm) = 12.86 (s, 2H), 7.92 (d, *J*_*H, H*_ = 8.0 Hz, 4 H, C*H*_ar_), 7.67 (d, *J*_*H, H*_ = 8.0 Hz, 4 H, C*H*_ar_), 7.48 (s, 2H, C*H*_th_), 2.84 (t, *J*_*H, H*_ = 7.6 Hz, 4 H, C*H*_2_), 2.07 (m, 2H), 1.92 (s, 6H).

### Synthesis of Mn-Doped CdS-ZnS Core-Shell QDs

Mn-doped CdS-ZnS core-shell QDs were prepared following the reported method with minor modifications (Yang et al., 2008). First, CdS core QDs were prepared using a direct heating-up method. In a typical synthesis, cadmium myristate (1.0 mmol), sulfur (0.5 mmol), and ODE (50 mL) were added to a 100 mL-flask. The resulting mixture was degassed at room temperature for 10 min, and then was heated to 240°C under N_2_ flow. The reaction was stopped by removing heating mantle and cooling to room temperature. The resulting CdS QDs were precipitated with addition of acetone, following by centrifugation and then were redispersed in hexane. A small amount of OLA can be added to assist QDs to disperse in hexane. The CdS QDs were dispersed in hexane as a stock after purified for three times. Second, ZnS shells were grown on the CdS cores using a layer by layer injection method. Typically, CdS cores (100 nmol) were mixed with ODE (3 mL) and OAm (1 mL), and the resulting mixture was degassed for 1 h at room temperature and then heat up to 220°C under N_2_ flow for ZnS growth. Zinc-stearate in ODE (0.1 M) and sulfur in ODE (0.1 M) were injected simultaneously for each monolayer growth of ZnS shell. The reaction time was 10 min after each injection. The growth for first two monolayers ZnS shells was at 220°C and then was at 280°C for later growth. Third, dopant growth was performed in the same pot with ZnS shells growth. Freshly made Mn(OAc)_2_ solution (5 mM) was injected after five monolayers of ZnS shells growth, accompanying the injection of sulfur precursor for the six monolayer. Dopant growth was allowed for 20 min and was followed by the growth of two more ZnS shells. Finally, zinc-stearate solution for the last monolayer ZnS shell growth (0.1 M, 2 mL) was injected and then the QDs were annealed at 240°C for 30 min. The resulting Mn-doped QDs were purified by three precipitation-redispersion cycles using acetone and hexane.

## Results and Discussions

**Figure 1:**
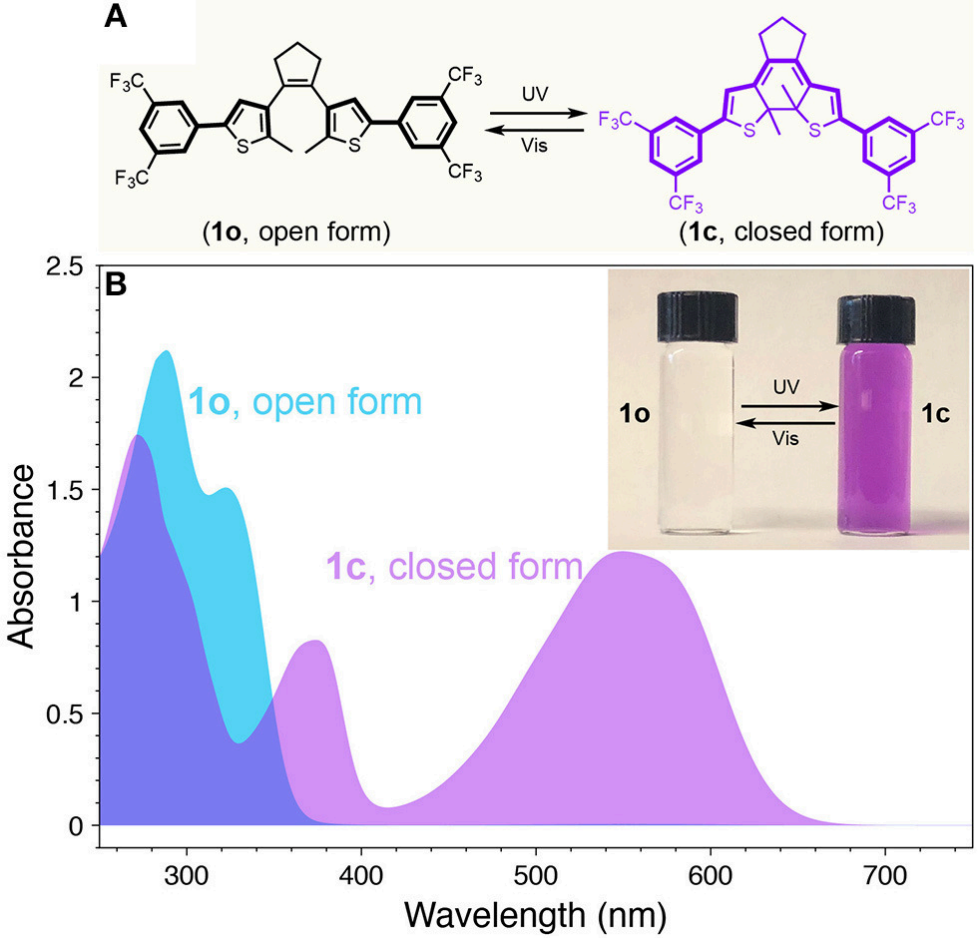
**(A)** Chemical structures and photoisomerization of diarylethene **1** (Vis = visible). **(B)** UV-vis absorption spectra of **1** at open (**1o**) and closed (**1c**) forms. Inset: photograph for the THF solution of **1o** (left) and **1c** (right).

We have synthesized diarylethene switch molecule featuring with electron withdrawing group of 3,5-bis(trifluoromethyl)benzene (**1**) as shown in Figure 1A. The ring-open isomer (**1o**) and ring-closed isomer (**1c**) can be interconverted by illumination with UV and visible light showing drastically different absorption feature. Specifically, compared to the open form (**1o**), whose π-conjugation is restricted to each half of the molecule, the closed form (**1c**) possesses extended π-conjugation across the entire molecule. This extended conjugation places the highest occupied molecular orbital (HOMO) and lowest unoccupied molecular orbital (LUMO) of the molecule closer, thus allowing the absorption profile red-shift correspondingly (Kuhn, 1949). UV-visible absorption spectra of the open and closed isomers (**1o** and **1c**) of diarylethene **1** are shown in Figure 1B. The open form (**1o**) as prepared only shows absorbance at wavelengths shorter than 380 nm. After illumination with UV light, the molecule turns into the closed isomer (**1c**) and a pronounced absorption peak appears in the visible range (420–650 nm). It should be noted that there is minimal absorbance from both isomers (Figure 1B), indicating a nearly transparent window in the range of 400–420 nm. This photo-switchable absorption feature motivated us that a dynamic emitting architectural construct can be achieved when modulated with appropriate dual-color emitting fluorophores.

**Figure 2:**
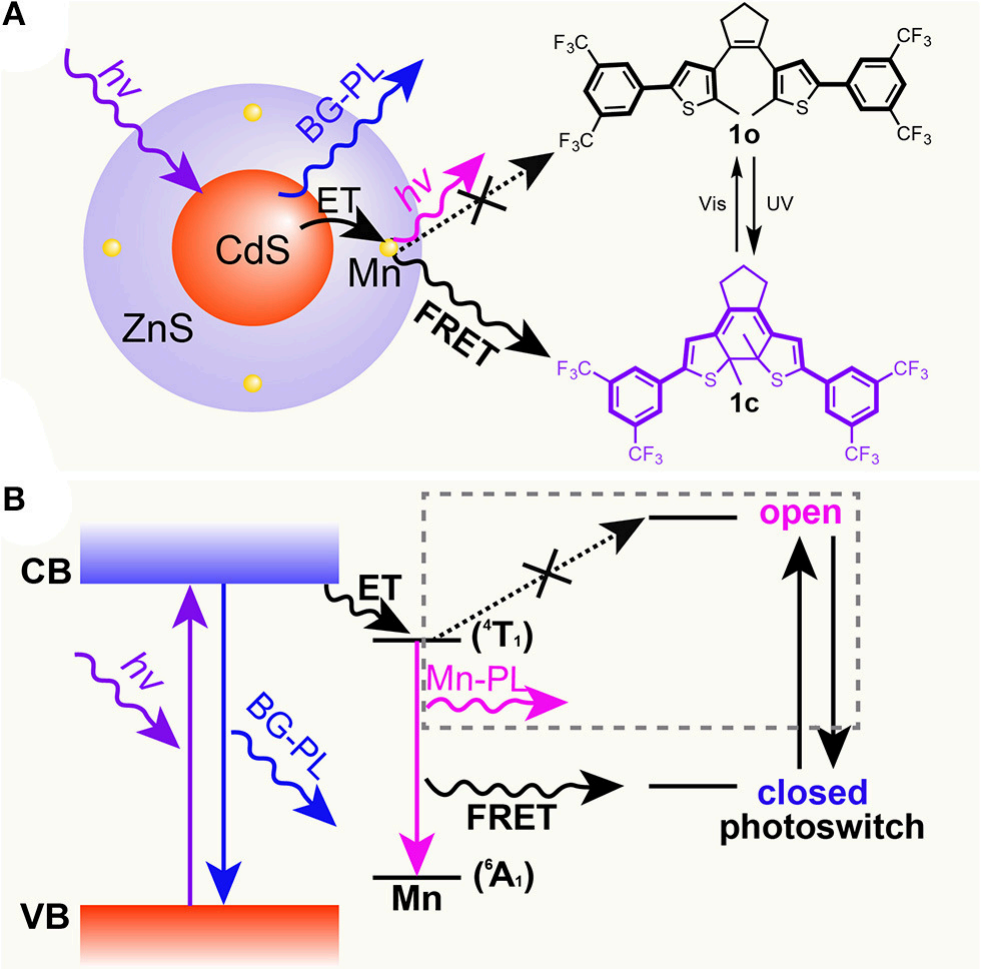
Scheme **(A)** and energy diagram **(B)** of proposed mechanism for the selective Mn-PL quenching between Mn-doped CdS-ZnS core-shell QDs and diarylethene **1** molecules.

As mentioned above, high-quality dual-color emitters can be obtained by doping transition metals or rare earth elements in QD lattices. Among them, Mn-doped CdS-ZnS QDs have been robustly synthesized and extensively studied (Yang et al., 2006, 2008, 2009; Chen O. et al., 2010; Chen et al., 2012). When excited with high energy photons, an electron-hole pair (an “exciton”) is created and confined inside the Mn-doped QD. This exciton can be radiatively deactivated through either recombination at the CdS-ZnS core-shell QD band edge to give a corresponding blue BG emission, or energy transfer to Mn dopants and subsequently emit a lower energy photon from the ^4^T_1_ to ^6^A_1_ states of the Mn ions (Figure 2) (Yang et al., 2009; Chen H.Y. et al., 2010). Most importantly, their dual-color emission bands can be adjusted to match the dynamic absorption windows of the diarylethene **1** (Figure 1) (Ithurria et al., 2007; Chen O. et al., 2010; Chen et al., 2012). Therefore, we hypothesize that when mixing the well-designed Mn-doped CdS-ZnS core-shell QDs and the photo-switchable diarylethene molecules together, FRET from the excited state (i.e., ^4^T_1_) of Mn to the switches can be turned on and off in the closed (**1c**) and open (**1o**) forms, respectively (Figure 2). Thus, the system would show a combined color of both BG- and Mn-PL of the QDs while the diarylethene **1** stays at the open form (**1o**) and show the emission color mostly from BG-PL when the diarylethene **1** turns into the closed form (**1c**) (Figure 2). This color switching process could be simply modulated by illuminating with UV or visible light.

**Figure 3:**
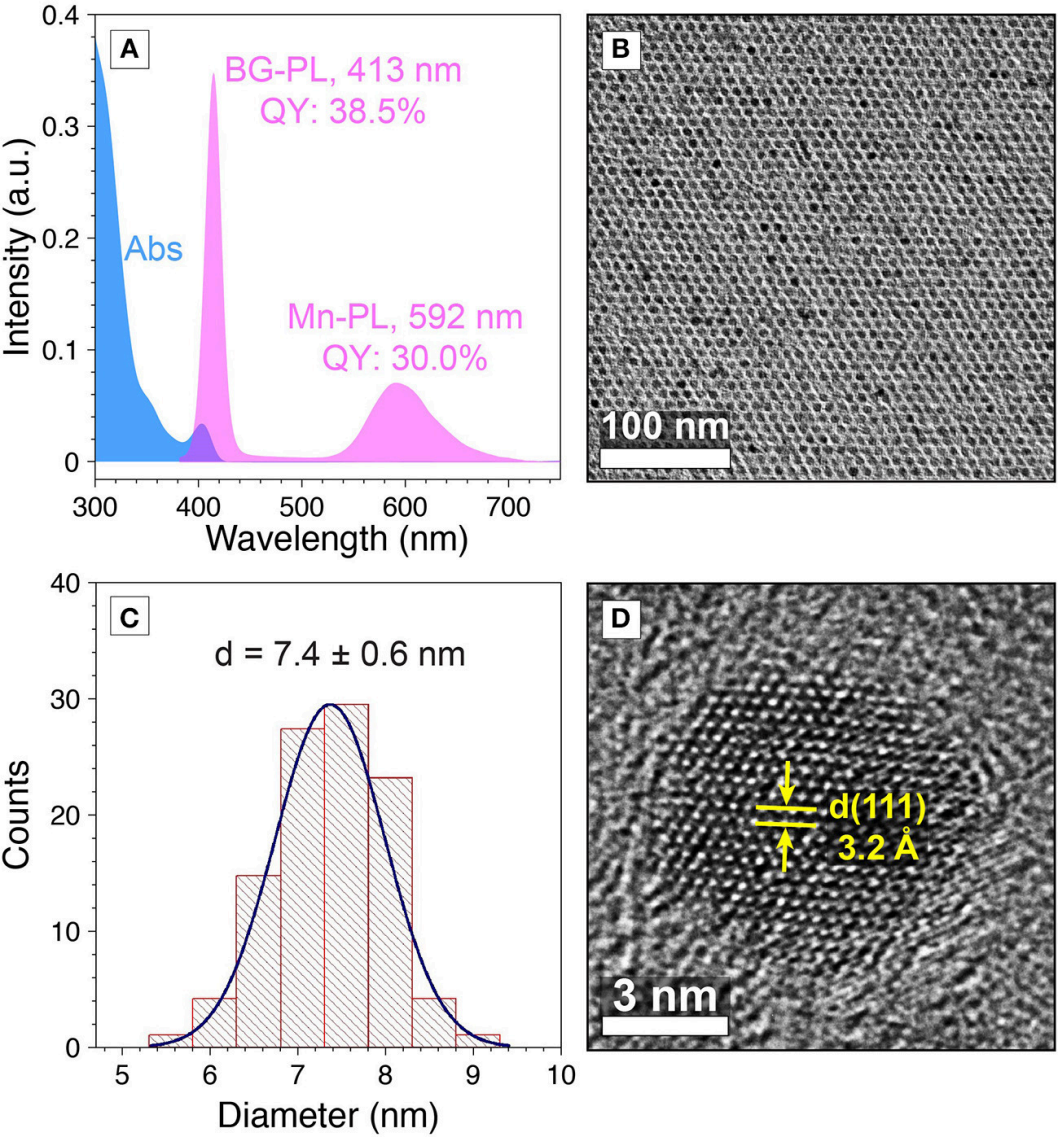
**(A)** Absorption (blue) PL (pink) spectra, and **(B)** TEM image of Mn-doped CdS-ZnS core-shell QDs. **(C)** Size distribution histogram of Mn-doped CdS-ZnS core-shell QDs. **(D)** HR-TEM image for a Mn-doped CdS-ZnS core-shell QD.

To test our hypothesis, we designed and synthesized Mn-doped CdS-ZnS core-shell QDs with desired emission bands to match with the absorption profiles of the diarylethene **1**. In particular, the BG-PL centered at 413 nm with a full width at half maximum (FWHM) of 16 nm (PL QY of 38.5%) was achieved by controlling the CdS core size and ZnS shell thickness (see materials and methods for details) (Figure 3A). This BG-PL lays in the transmission window of the switch molecule disregarding to the open or closed form (Figure 1B). Meanwhile, in order to achieve a large spectral overlap between the Mn-PL and absorption feature of the **1c**, Mn-PL centered at 592 nm (PL QY of 30.0%) was accessed by doping Mn ions closer to the surface of the QDs, thus minimizing the local strain of Mn impurities (Figure 3A) (Ithurria et al., 2007). The Mn-to-BG PL intensity ratio was determined to be 0.24 (Figure 3A). Transmission electron microscopy (TEM) measurements showed the resultant QDs exhibited a spherical shape and a high morphological uniformity with an average diameter of 7.4 ± 0.6 nm (Figures 3B, C). High-resolution TEM (HR-TEM) image showed (111) plane with *d*-spacing of 3.2 Å (Figure 3D).

**Figure 4:**
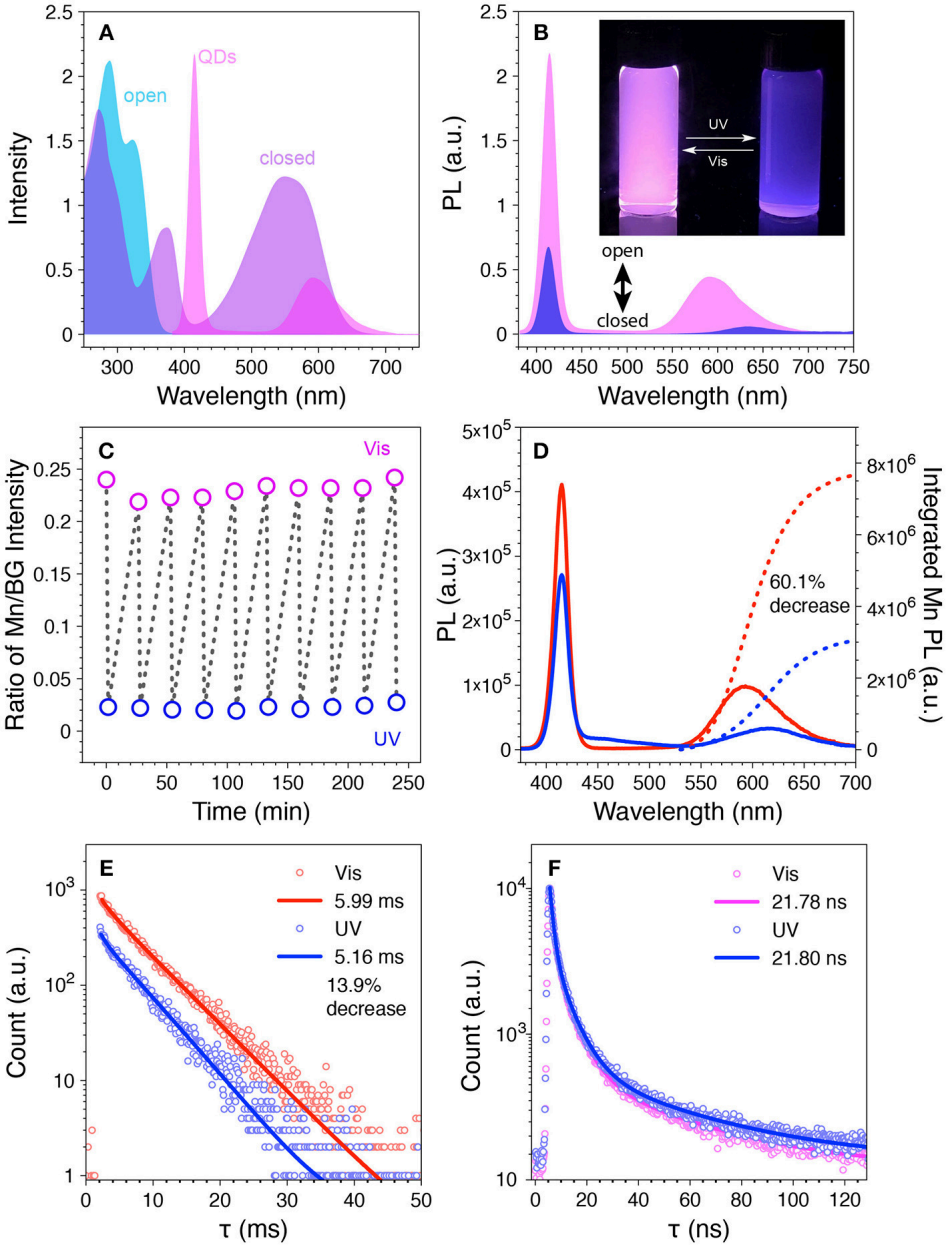
**(A)** Overlay of the PL spectrum of the Mn-doped CdS-ZnS QDs with the absorption spectra of the **1o** and **1c**. **(B)** PL spectra of the mixture of Mn-doped CdS-ZnS QDs with **1o** (pink) and **1c** (blue). Inset: photograph for the mixture solution under UV light after UV (right) and visible (left) light illumination. **(C)** The ratio of Mn/BG-PL intensity during repetitive switching cycles with sequential UV (blue open circle) and visible (pink open circle) light illumination. **(D)** The PL intensity (solid lines) and integrated Mn-PL intensity (dotted lines) and **(E)** the corresponding Mn-PL lifetime changes during one switching cycle. **(F)** BG-PL lifetime decays after UV (blue) and visible (pink) light illumination.

The prepared Mn-doped CdS-ZnS QDs and diarylethene **1** were employed to test the photo-switching property. It is shown that while both the BG- and Mn-PL are disjoint from the absorbance of **1o**, the BG-PL locates at the absorbance depression area and most of the Mn-PL overlaps with visible-range absorbance of **1c** (Figure 4A). We first tested the Mn-doped CdS-ZnS QDs under illumination of either UV or visible light without mixing with diarylethene **1** switches. Both BG- and Mn-PL intensities remained without any variation, indicating the robustness of the QD samples under light illumination. However, when mixing the same QDs with **1o** at a molar ratio of 1:500 in THF, the QD solution showed a pink color from the intact emission due to a minimal spectral overlap between the QD emission and the **1o** absorbance (Figures 4A, B). When illuminated with UV light (365 nm) for 90 s, a strong absorption feature arose in the visible range (420–650 nm, Supplementary Figure 2a), accompanying the decrease of the ratio of Mn/BG PL intensities from 0.24 to 0.02, corresponding to a change of the emission color from pink to blue (Figure 4B and Supplementary Figure 2b). The change of the PL intensity ratio is largely due to the dynamic Mn-PL quenching and recovering effect when the switch molecule alternates between closed (**1c**, quenching) and open (**1o**, recovering) forms (Figure 4B). Importantly, this observed photo-switching process is highly reversible. Ten photo-switching cycles were carried out to test the fatigue resistance of the switch molecule (i.e., diarylethene **1**), and the stability of the entire system. During these cycles, both the absorption spectra and the intensity ratios of the Mn/BG PL peaks showed excellent reversibility (Figure 4C and Supplementary Figures 3, 4), indicating a reliable photo-switching property of our designed system.

Two possible Mn-PL quenching/recovering mechanisms are attributable to the observed photo-switching property: 1) FRET from the excited state (i.e., ^4^T_1_) of Mn dopant ions to the **1c** non-radiatively; 2) radiated photons of Mn-PL can be reabsorbed by the surrounding **1c**. To explore the origin of the observed Mn-PL quenching effect and their contributions, time-resolved PL lifetime measurements were carried out at different stage of the photo-switching process. When the integrated intensity of the Mn-PL decreased 60.1% of its initial value, the Mn-PL lifetime decreased from 5.99 to 5.16 ms, indicating that a 13.9% of the Mn-PL quenching can be attributed to FRET for the Mn-PL to the switch **1c** molecule in the measuring conditions (Figures 3, 4D,E, see Supplementary Material for detailed calculation) (Medintz et al., 2003; Gu et al., 2008; Niebling et al., 2009; Tu et al., 2011; Krivenkov et al., 2019). Consequently, the rest 46.2% of Mn-PL quenching can be explained by the radiative photon reabsorption by surrounding **1c** molecule. A 31.0% of BG-PL integrated intensity decrease was also observed which again was contributed to the photon reabsorption due to the slight increase of the absorbance from the switch molecule (from **1o** to **1c**). No variance in PL lifetime (~21 ns) of the BG-PL further confirmed the reabsorption process (Figure 4F and Supplementary Figure 5) without influencing the radiative photon recombination dynamics (Medintz et al., 2003). It is known that the efficiency of FRET process is strongly sensitive to the distance between donors and acceptors (inverse sixth power of the distance between donor and acceptor, typically within 1–10 nm) (Selvin, 2000). FRET process can happen only when the **1c** molecules reach to a close proximity (<10 nm) of the QD surface, which is in line with our experimental observation that only a small portion (13.9% of the total quenching) of the Mn-PL quenching is caused by FRET. Moreover, given the size of the QDs (i.e., 7.4 nm) and the doping radial location of the Mn dopants inside the QDs (at the interface between the 5th and the 6th monolayer of the ZnS shell), the distances between the photo-generated exciton (electron-hole pair) center and the **1c** is 3.2 nm larger than that between the Mn ion and the **1c** the exciton center is determined at the center of core-shell QDs, (Yang et al., 2009). This larger distance will dramatically decrease the FRET efficiency between the exciton and **1c** as compared to that from Mn ion to **1c**. This again agrees well with the fact that the decrease of BG-PL is mostly attributable to the photon reabsorption process.

**Figure 5:**
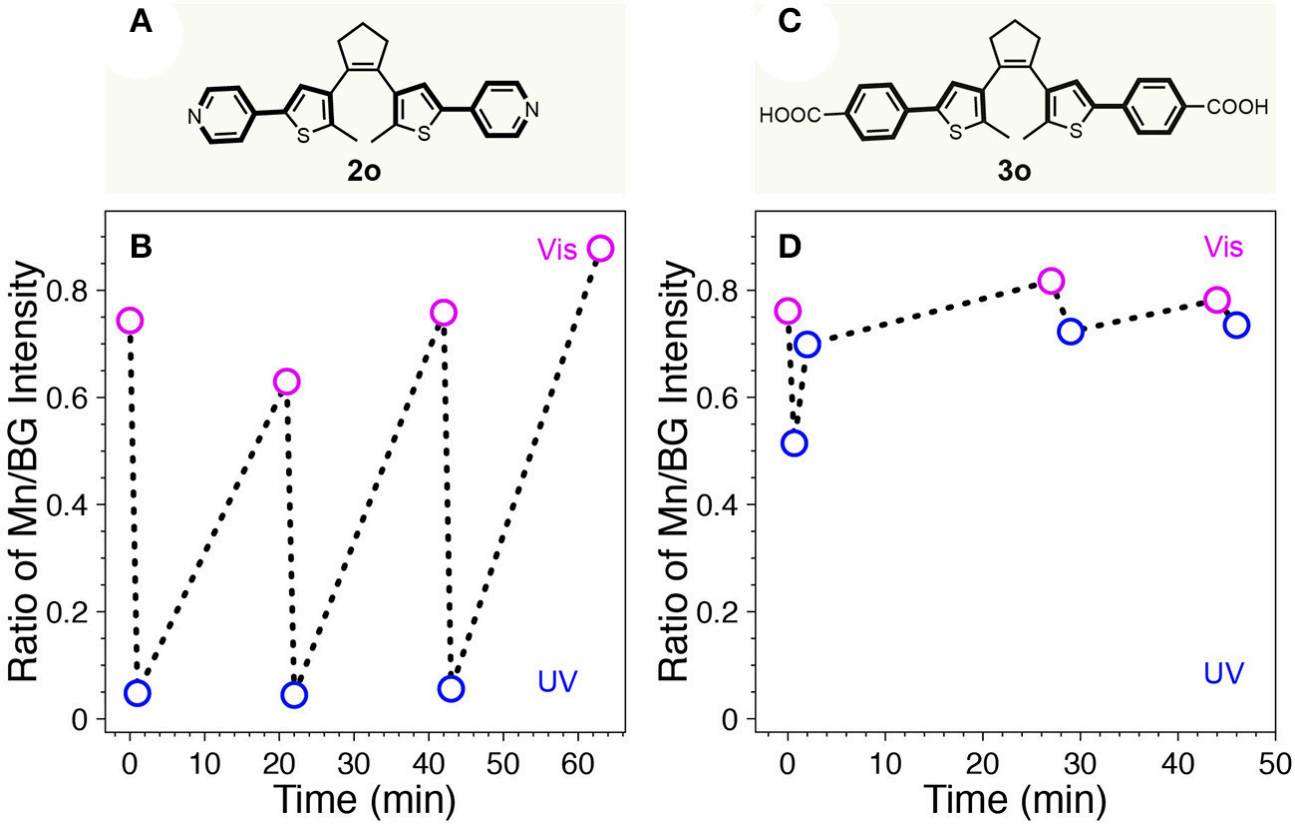
Chemical structures of **2o (A)** and **3o (C)**. The ratio of Mn/BG-PL intensity of the mixture of Mn-doped CdS-ZnS QDs with **2o (B)** or **3o (D)** during repetitive switching cycles with sequential UV (blue open circle) and visible (pink open circle) light illumination.

Recently, it was reported that a series of diarylethene switch molecules with electron withdrawing substituents on the adjacent phenyl rings could provide fatigue resistance due to minimized formation of annulated isomers (Herder et al., 2015). According to the study, two other diarylethene-based switches terminated with electron withdrawing groups of pyridine (diarylethene **2**) and 4-benzoic acid (diarylethene **3**) were synthesized and tested in our system (Figure 5). Diarylethene **2** was mixed with Mn-doped CdS-ZnS core-shell QDs possessing BG- and Mn-PL peaks at 414 and 600 nm, respectively. The absorption spectral evolution clearly indicated fatigue effect over only 3 cycles (Supplementary Figure 6). Accordingly, the BG- and Mn-PL were quenched after visible light illumination whereas the quenching effect was reduced after UV irradiation after cycles (Supplementary Figure 7). Since the BG-PL was more affected by the fatigue of diarylethene **2**, the ratio of Mn/BG intensity increased with 3 cycles (Figure 5B). Diarylethene photo-switch **3**, which possesses carboxylic groups at the para position of the phenyl rings, was expected to attach on the surface of QDs covalently. This direct attachment of the switch **3** molecules would significantly reduce the mean distance between QDs donor and switch acceptor, thus facilitate the FRET from QD to the **3** molecules. However, our result showed that while **3o** itself can be isomerized to the closed form (**3c**) under UV illumination, it irreversibly stays at its open form (**3o**) mostly after mixing with Mn-doped CdS-ZnS QDs (Supplementary Figure 8). It is shown that while the Mn/BG PL intensity ratio decreased with 40 s of UV illumination, it increased back with further UV illumination (Figure 5D). This phenomenon can be ascribed to the direct attachment of switch **3** to the surface of the QDs through the carboxylate functional group, thus leading to a close proximity of switch **3** to the QD surface constantly rather than a dynamic on-and-off process as for switches **1** and **2** cases. In this case, the visible Mn-PL of the QDs can trap the switch molecule **3** in its open form (**3o**), preventing them from turning into the closed form (**3c**). Consequently, no reversible photo-switching phenomenon can be observed as shown in Figure 5D, in good agreement with our experimental observations.

## Conclusions

To conclude, we demonstrate a photo-switchable hybrid system with a reliable dual-color performance. This system combines photo-switchable diarylethene **1** molecule functionalized with strong electron withdrawing group of 3,5-bis(trifluoromethyl)benzene, and Mn-doped CdS-ZnS QDs with dual-emission band. Selective quenching/recovering of Mn-PL was achieved effectively, resulting in a pink and blue dual-color switching behavior under UV and visible light illumination. This photo-switching process is highly reversible and shows superior fatigue-resistance for at least 10 switching cycles. The mechanism studies using both steady-state and time-resolved PL spectroscopy reveal the PL quenching contributions from both FRET and photo-reabsorption processes. Moreover, we show that the involvements of other electron withdrawing functional groups (i.e., pyridine and carboxylate groups) limit the photo-switching property by significant molecular fatigue or irreversible optical effects. Our study sheds light on the fabrications of highly dynamic and photo-switchable hybrid systems that hold the potential in a broad range of applications spanning from self-erasing paper, biological-imaging to single molecule sensing/tracking and super-resolution imaging/localization microscopy.

## Data Availability

The datasets for this manuscript are not publicly available because can be available upon request. Requests to access the datasets should be directed to ouchen@brown.edu.

## Author Contributions

YY, HZ, AD, and WZ performed experiments. YY, RT, and OC designed experiments. YY, YN, and OC interpreted the data and wrote the paper.

### Conflict of Interest Statement

The authors declare that the research was conducted in the absence of any commercial or financial relationships that could be construed as a potential conflict of interest.
