# Supplementary material for: Reversible Photo-Switching of Dual-Color Fluorescent Mn-Doped CdS-ZnS Quantum Dots Modulated by Diarylethene Molecules
Source: Front Chem. 2019 Mar 20;7:145. doi: 10.3389/fchem.2019.00145 (PMC6435480; doi:10.3389/fchem.2019.00145)
Supplement: Supplementary file 1 [file Data_Sheet_1.pdf]

Supplementary Material

Reversible Photo-Switching of Dual-Color Fluorescent Mn-Doped CdS-ZnS Quantum Dots Modulated by Diarylethene Molecules

Yucheng Yuan[1], Hua Zhu[1], Yasutaka Nagaoka[1], Rui Tan[1], Andrew Hunter Davis[2], Weiwei Zheng[2], Ou Chen[1*]

[1]Department of Chemistry, Brown University, Providence, Rhode Island 02912, United States

[2]Department of Chemistry, Syracuse University, Syracuse, New York 13244, United States

*** Correspondence:**
Ou Chen
ouchen@brown.edu

1 Supplementary Experimental Data

1.1 Synthesis of 1,2-Bis(5-chloro-2-methylthiophen-3-yl)cyclopent-1-ene (Supplementary Figure 1)

The synthesis of 1,2-Bis(5-chloro-2-methylthiophen-3-yl)cyclopent-1-ene followed previous reported method with minor modifications (Park, Jiang et al. 2016). To an ice-cooled mixture of 2-chloro-5-methylthiophene (298 mmol) and glutaryl dichloride (150 mmol) in CS_2, $AlCl_3$ (360 mmol) was added under vigorous stirring. Then the mixture was stirred for 2 hrs at room temperature. Afterwards, ice-cold water (100 mL) was added to the reaction mixture and the resulting solution was extracted with CH_2Cl_2 (3 × 150 mL). The combined organic layer was washed with brine (100 mL) and then dried over Na_2SO_4. Then the solvent was evaporated to yield 1,5-Bis-3-(2-chloro-5-dimethylthienyl)-1,5-pentadione as a brown tar and used for next step without further purification.

THF (50 mL) and Zn dust (38 mmol) were added to a three-neck flask under N_2 flow. Then $TiCl_4$ (29 mmol) was injected cautiously with syringe. The solution turned yellow and was reflux for 45 min. After cooled using an ice bath, 1,5-Bis-3-(2-chloro-5-dimethylthienyl)-1,5-pentadione (19.2 mmol) was added and the reaction mixture was reheated and refluxed for 4 hrs. 10% K_2CO_3 solution (50 mL) was added to the mixture followed by filtration over Celite and washing with EtOAc (3 × 20 mL), and the filtrate was dried over anhydrous Na_2SO_4. After the solvent was evaporated, the crude product was purified by column chromatography (silica, pure hexanes) to afford 1,2-Bis(5-chloro-2-methylthiophen-3-yl)cyclopent-1-ene as a white solid.

1.2 UV-Vis Absorption Measurements

UV-Vis absorption spectra were measured using an Agilent Technologies Cary 5000 UV-Vis Spectrophotometer. QDs and diarylethenes were dissolved in THF for the measurements.

1.3 Fluorescence Spectroscopy

The solution photoluminescence (PL), lifetime and quantum yield (QY) measurements were performed on an Edinburgh Instruments Fluorescence Spectrometer FS5. THF was used as solution for measurements. The PL lifetime were measured with excitation at 360 nm, with an average acquisition time of 15 min. The PL QYs were measured by FS5 Spectrometer with a built in integrating sphere. In the photoswitchable study, a UltraBright UV Transilluminator (MLB-16, Maestrogen) and a mounted 590 nm LED (M590L3, Thorlabs) were used as irradiation light source.

1.4 TEM Measurements

TEM measurements were performed on a JEOL 2100F operated at 200 kV. The QDs were diluted in hexane after purification, then drop cast on a 300-mesh copper TEM grid and dried at ambient condition before TEM measurements.

1.5 NMR Measurements

^{1}H NMR spectra were recorded on a Bruker ARTEMIS instrument. Chemical shifts values are given in ppm and are referred to $CDCl_3$: 7.26 ppm. Data are reported as follows: chemical shift, multiplicity (s = singlet, d = doublet, t = triplet, br = broad, m = multiplet). The coupling constants *J*, are reported in Hertz (Hz).

1.6 Calculation of FRET Efficiency and Reabsorption

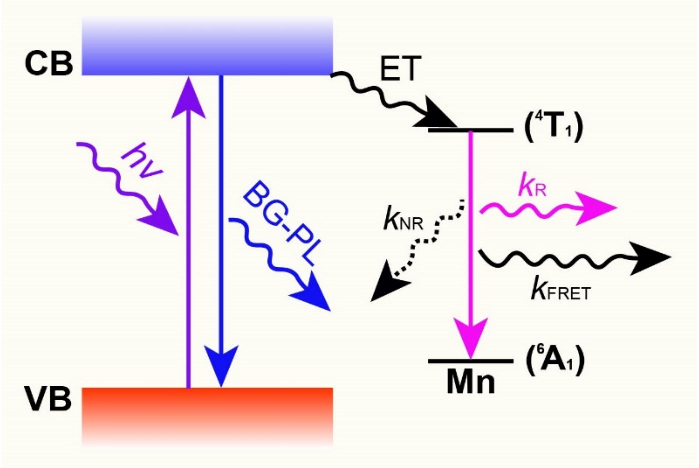

The simple model of the Mn-doped CdS-ZnS QDs is as illustrated above. Without mixing with diarylethene molecules, there are two processes occurring in the Mn relaxation, which are radiative decay with the rate constant k_R, and non-radiative decay with the rate constant k_{NR}. The Mn-PL QY (QY_1) and lifetime (τ_1) can be expressed as the following equations:

$$QY_1 = \frac{k_R}{k_R + k_{NR}} \quad (1)$$

$$\tau_1 = \frac{1}{k_\mathrm{R}+k_\mathrm{NR}} \tag{2}$$

When mixed with diarylethene molecules, a new nonradiative transfer (i.e. FRET) occurs, with the rate constant k_FRET. The corresponding Mn-PL QY (QY_2, note the QY here is without considering reabsorption) and lifetime (τ_2) can be expressed as:

$$\mathrm{QY}_2 = \frac{k_\mathrm{R}}{k_\mathrm{R}+k_\mathrm{NR}+k_\mathrm{FRET}} \tag{3}$$

$$\tau_2 = \frac{1}{k_\mathrm{R}+k_\mathrm{NR}+k_\mathrm{FRET}} \tag{4}$$

The FRET efficiency (Φ_FRET) can be expressed as:

$$\Phi_\mathrm{FRET} = 1 - \frac{\mathrm{QY}_2}{\mathrm{QY}_1} = \frac{k_\mathrm{FRET}}{k_\mathrm{R}+k_\mathrm{NR}+k_\mathrm{FRET}} \tag{5}$$

It can also be expressed as

$$\Phi_\mathrm{FRET} = 1 - \frac{\tau_2}{\tau_1} = \frac{k_\mathrm{FRET}}{k_\mathrm{R}+k_\mathrm{NR}+k_\mathrm{FRET}} = 1 - \frac{5.16\ \mathrm{ms}}{5.99\ \mathrm{ms}} = 13.9\% \tag{6}$$

In the real case, the FRET process is accompanying with the reabsorption process, the measured QY decrease (60.1%) is a combination of Φ_FRET and reabsorption. So, the reabsorption fraction can be calculated as 60.1% − 13.9% = 46.2%.

2 Supplementary Figures and Tables

2.1 Supplementary Figures

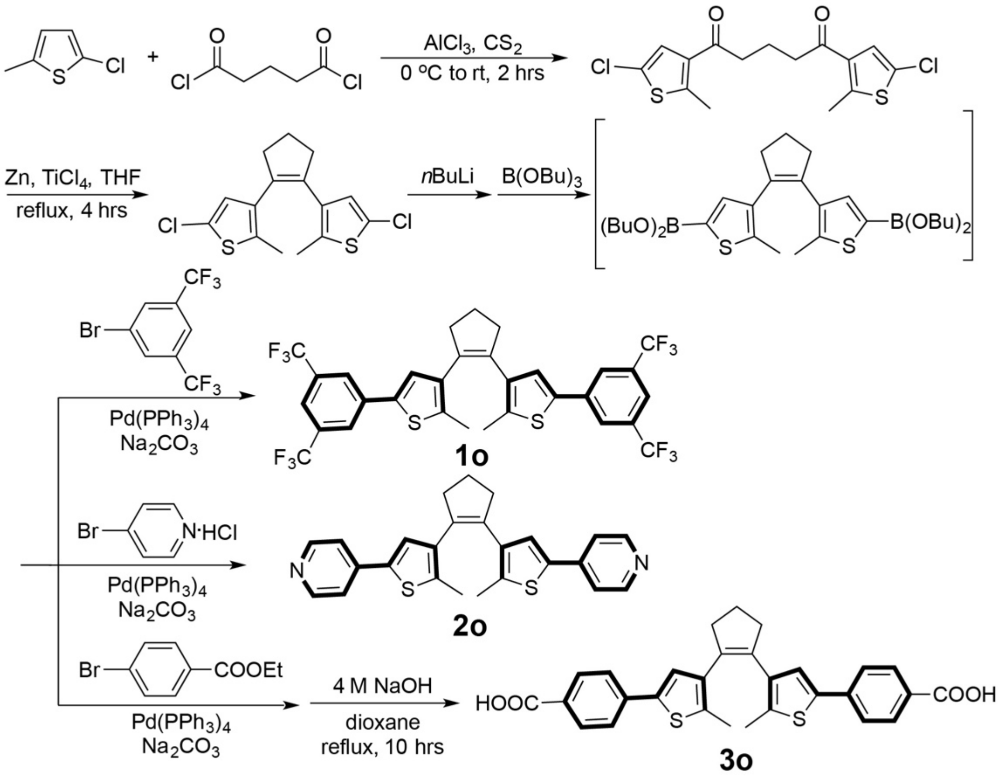

Supplementary Figure 1. Synthetic route for diarylethenes.

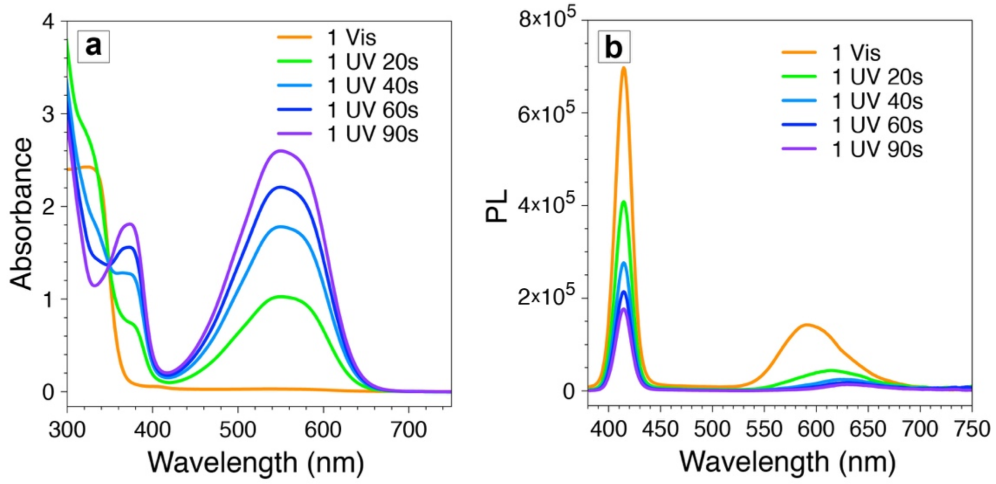

Supplementary Figure 2. Absorption (a) and PL (b) spectral evolution at different time in one cycle of mixture of Mn-doped CdS-ZnS QDs (BG-PL of 413 nm, Mn-PL of 592 nm) and diarylethene **1** in solution of THF under UV irradiation (365 nm).

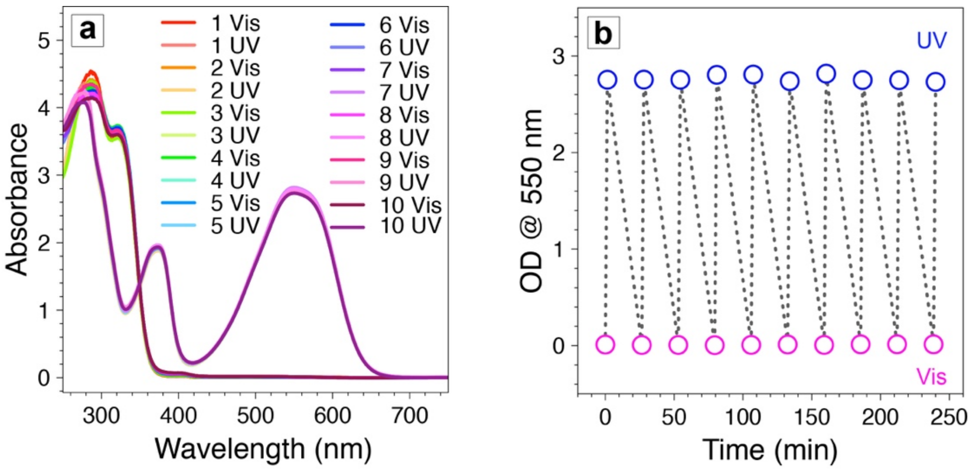

Supplementary Figure 3. Evolution of absorption property of mixture of Mn-doped CdS-ZnS QDs (BG-PL of 413 nm, Mn-PL of 592 nm) and diarylethene **1** in solution of THF for ten cycles. (a) Absorption spectra during repetitive switching cycles consisting of alternating UV (365 nm, 90 s) and visible irradiation (590 nm, 25 min). (b) Evolution of absorbance at the wavelength of 550 nm.

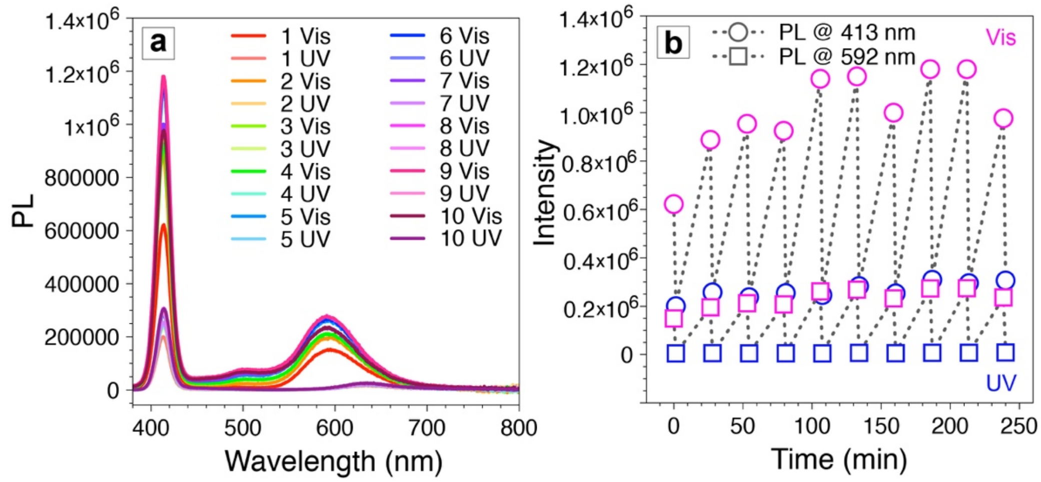

Supplementary Figure 4. Evolution of PL property of mixture of Mn-doped CdS-ZnS QDs (BG-PL of 413 nm, Mn-PL of 592 nm) and diarylethene **1** in solution of THF for ten cycles. (a) PL spectra during repetitive switching cycles consisting of alternating UV (365 nm, 90 s) and visible irradiation (590 nm, 25 min). (b) Evolution of PL at wavelength of 413 nm (open circle) and 592 nm (open square).

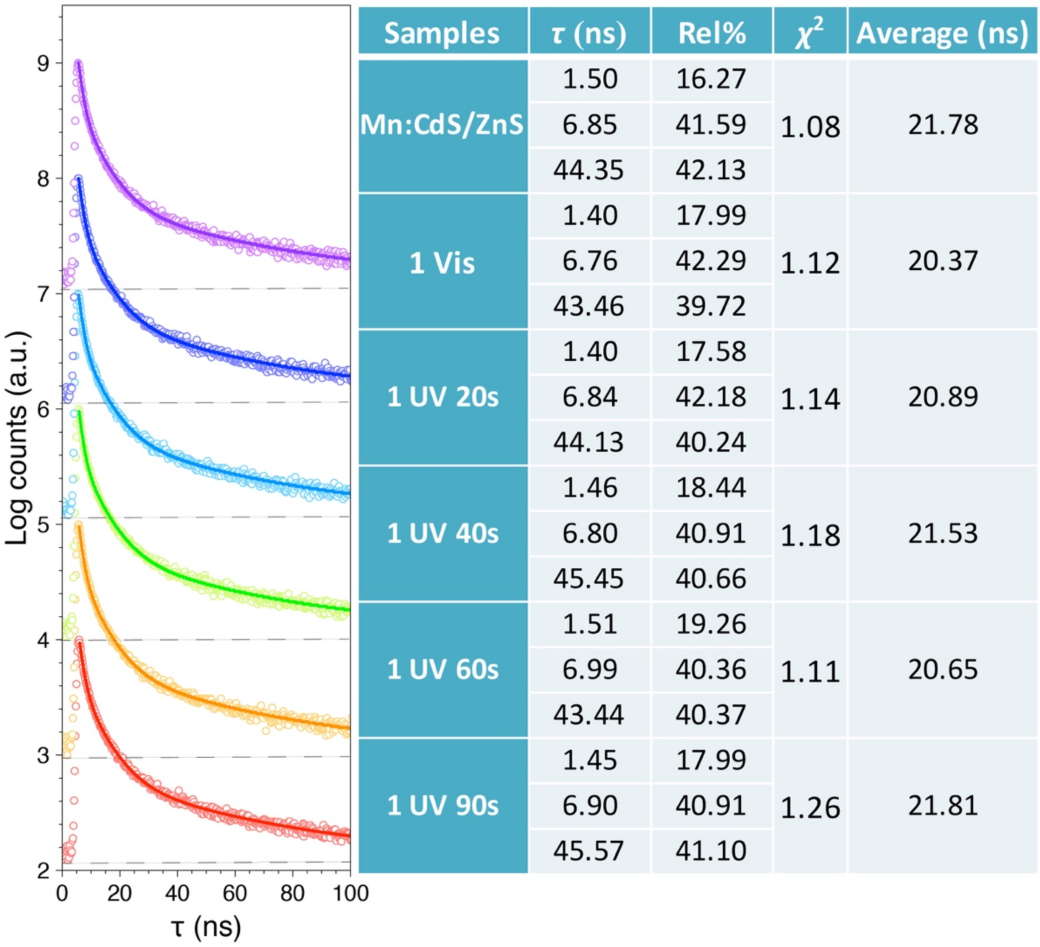

Samples	τ (ns)	Rel%	χ^2	Average (ns)
Mn:CdS/ZnS	1.50	16.27	1.08	21.78
	6.85	41.59		
	44.35	42.13		
1 Vis	1.40	17.99	1.12	20.37
	6.76	42.29		
	43.46	39.72		
1 UV 20s	1.40	17.58	1.14	20.89
	6.84	42.18		
	44.13	40.24		
1 UV 40s	1.46	18.44	1.18	21.53
	6.80	40.91		
	45.45	40.66		
1 UV 60s	1.51	19.26	1.11	20.65
	6.99	40.36		
	43.44	40.37		
1 UV 90s	1.45	17.99	1.26	21.81
	6.90	40.91		
	45.57	41.10		

Supplementary Figure 5. BG-PL lifetime evolution at different time in one cycle of mixture of Mn-doped CdS-ZnS QDs (BG-PL of 413 nm, Mn-PL of 592 nm) and diarylethene **1** in solution of THF under UV irradiation.

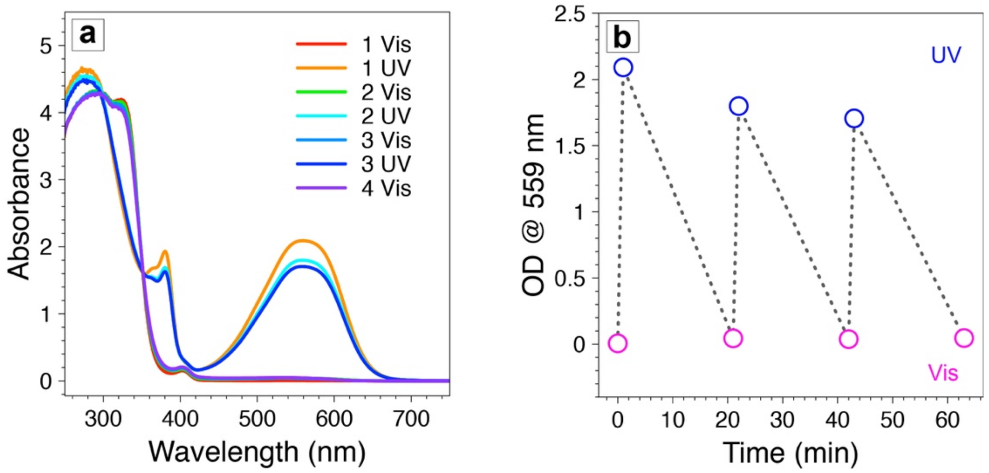

Supplementary Figure 6. Evolution of absorption property of mixture of Mn-doped CdS-ZnS QDs (BG-PL of 414 nm, Mn-PL of 600 nm) and diarylethene **2** in solution of THF for four cycles. (a) Absorption spectra during repetitive switching cycles consisting of alternating UV (365 nm, 90 s) and visible irradiation (590 nm, 25 min). (b) Evolution of absorbance at the wavelength of 559 nm.

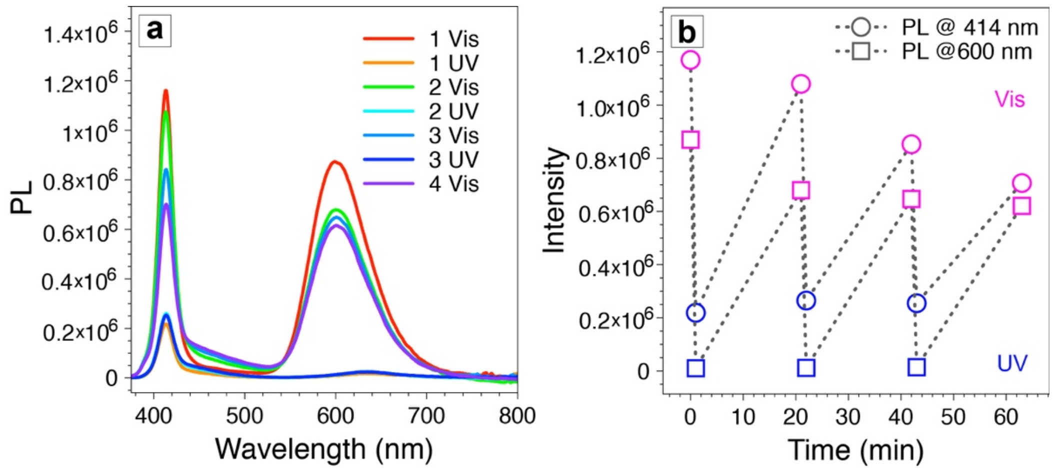

Supplementary Figure 7. Evolution of absorption property of mixture of Mn-doped CdS-ZnS QDs (BG-PL of 414 nm, Mn-PL of 600 nm) and diarylethene **2** in solution of THF for four cycles. (a) PL spectra during repetitive switching cycles consisting of alternating UV (365 nm, 90 s) and visible irradiation (590 nm, 25 min). (b) Evolution of PL at wavelength of 414 nm (open circle) and 600 nm (open square).

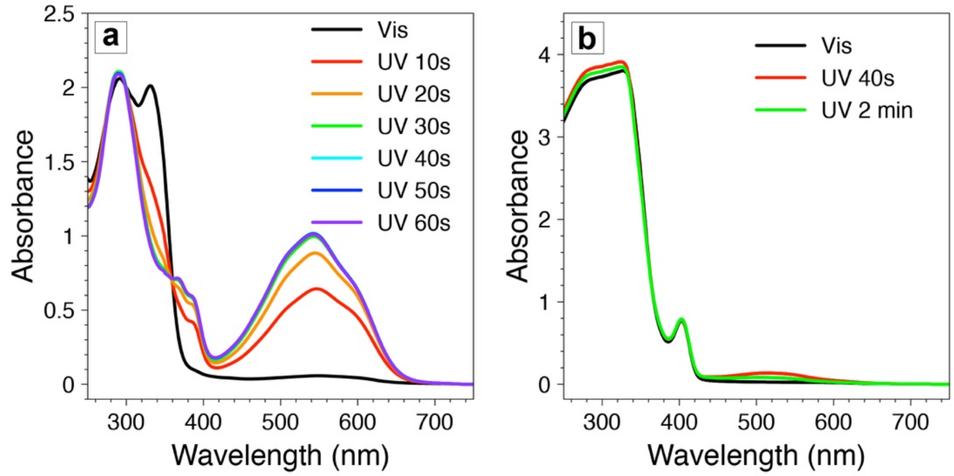

Supplementary Figure 8. (a) Absorption spectral evolution for diarylethene **3** at different time of UV irradiation (365 nm). (b) Absorption spectral evolution for the mixture of Mn-doped CdS-ZnS QDs (BG-PL of 414 nm, Mn-PL of 600 nm) and diarylethene **3** at different time of UV irradiation (365 nm).

2.2 ^{1}H NMR Spectra

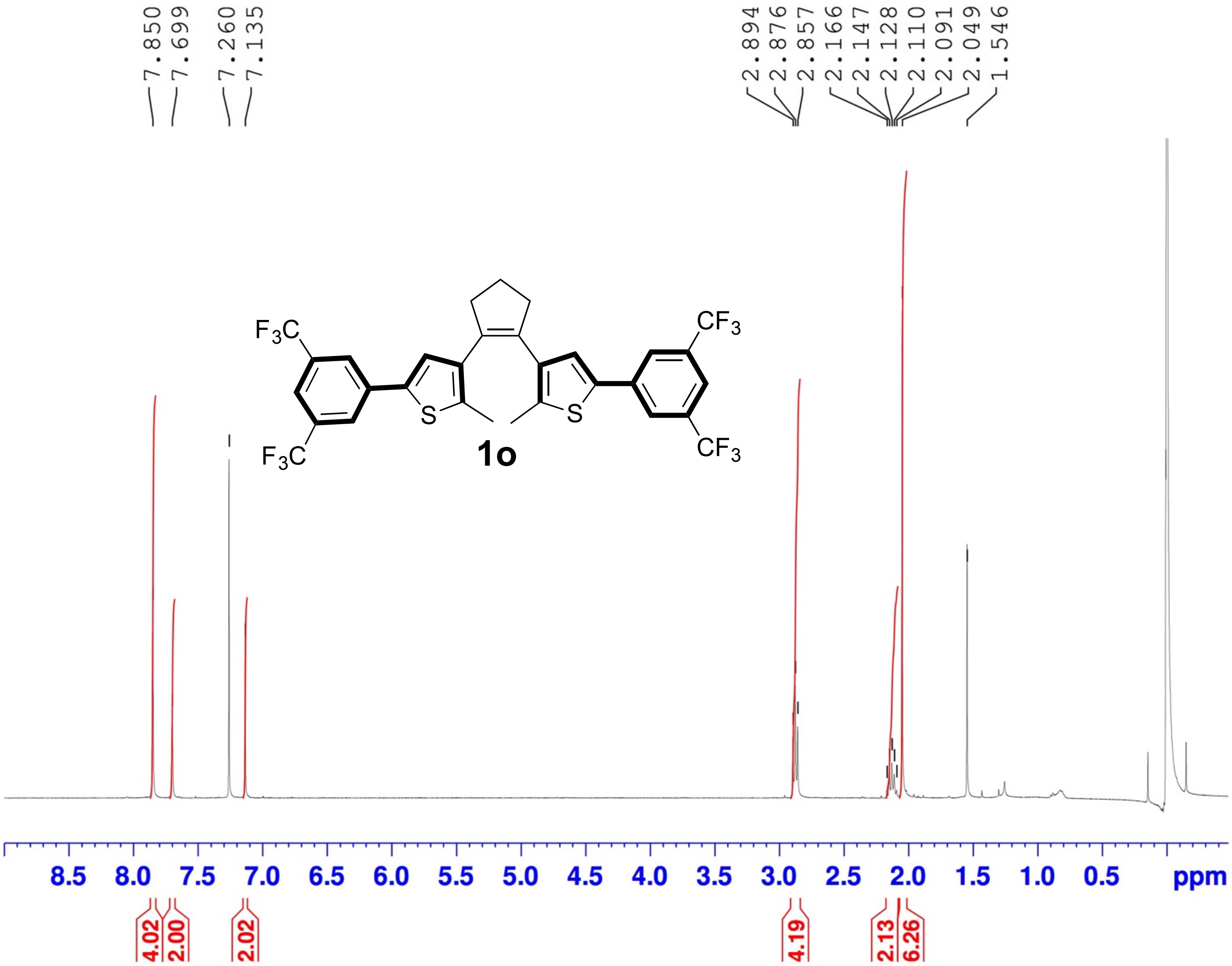

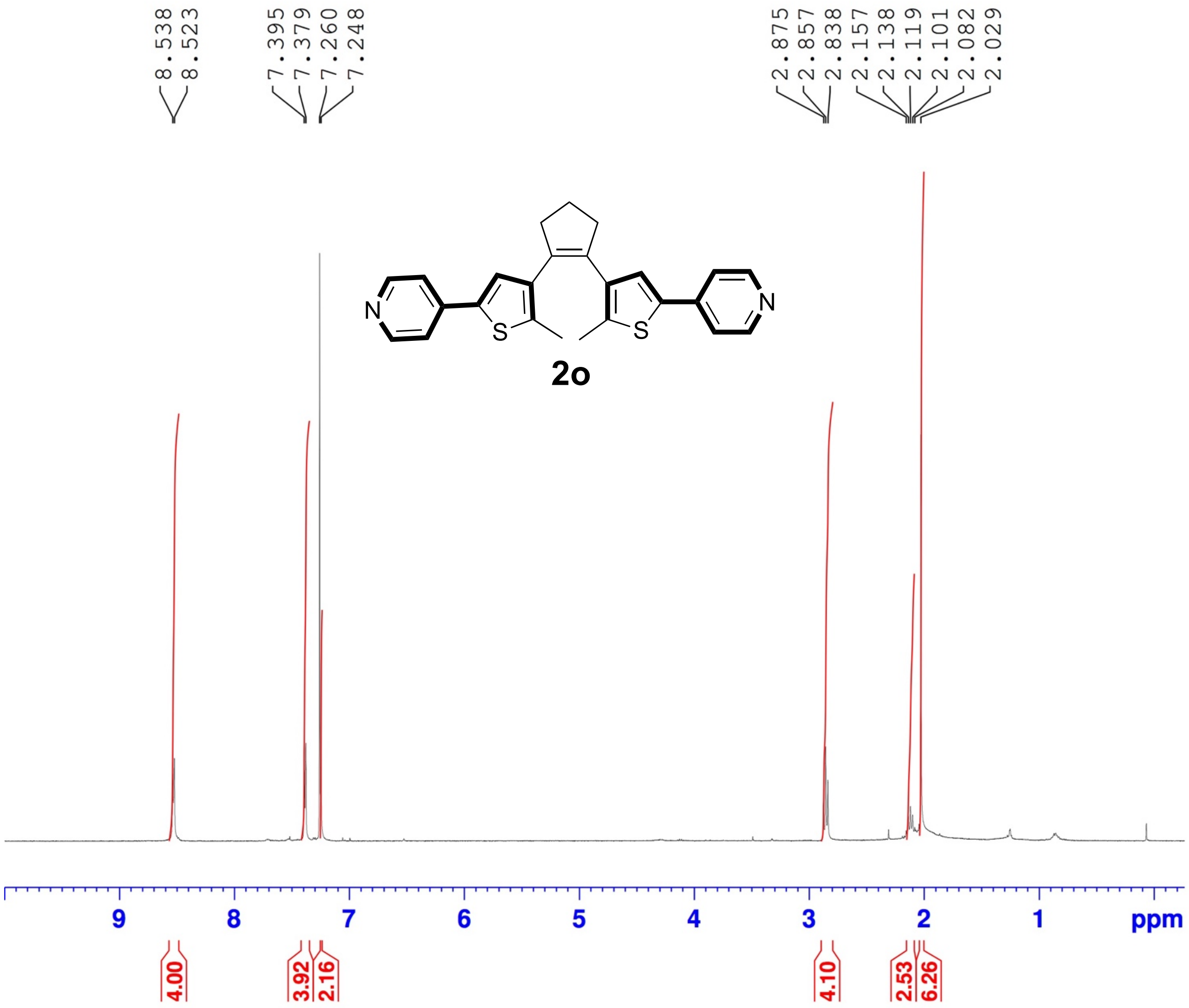
8.538
8.523
7.395
7.379
7.260
7.248
2.875
2.857
2.838
2.157
2.138
2.119
2.101
2.082
2.029
N
S
S
N
2o
9
8
7
6
5
4
3
2
1
ppm
4.00
3.92
2.16
4.10
2.53
6.26

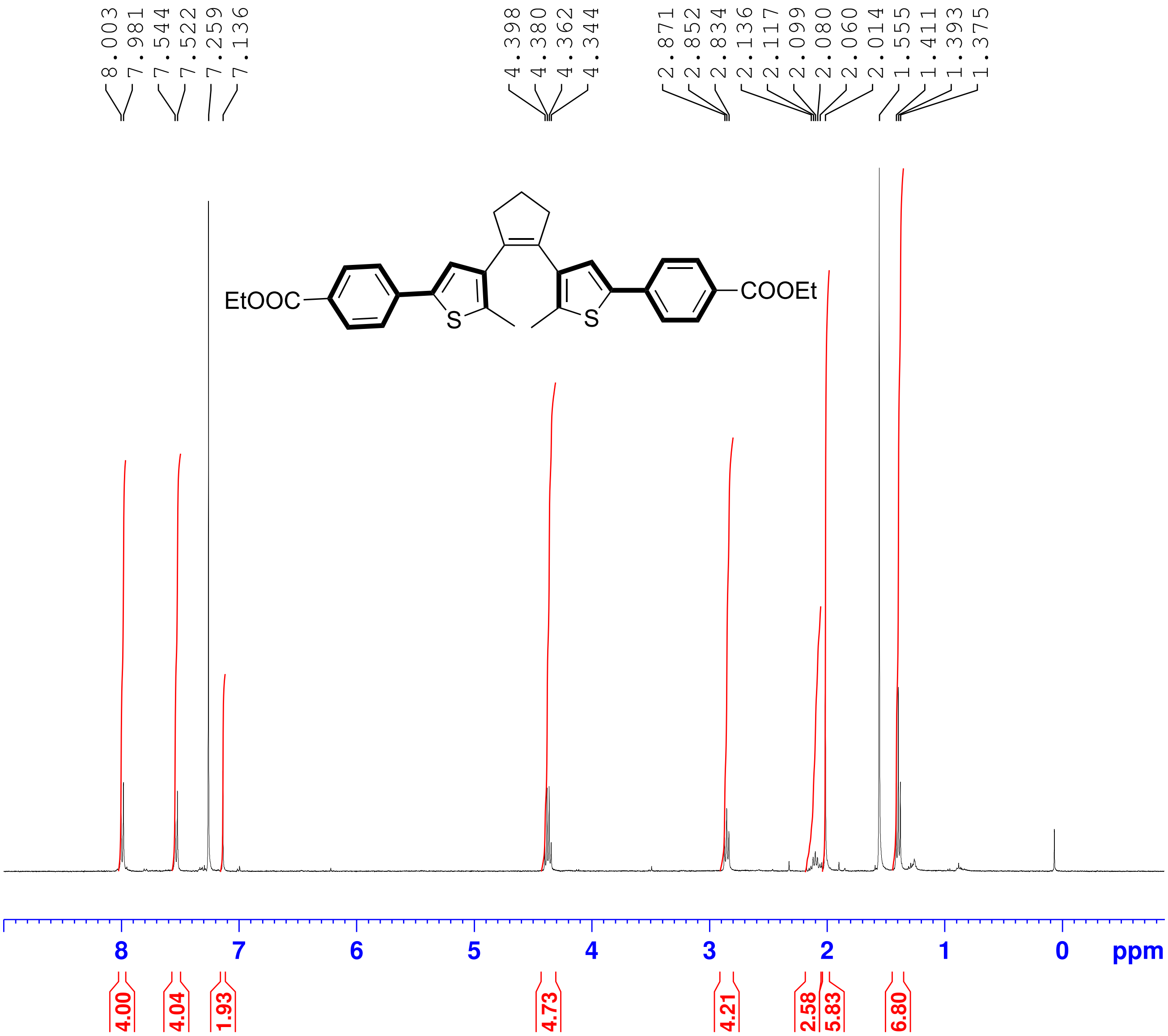
8.003
7.981
7.544
7.522
7.259
7.136
4.398
4.380
4.362
4.344
2.871
2.852
2.834
2.136
2.117
2.099
2.080
2.060
2.014
1.555
1.411
1.393
1.375
EtOOC
S
S
COOEt
8
7
6
5
4
3
2
1
0
ppm
4.00
4.04
1.93
4.73
4.21
2.58
5.83
6.80

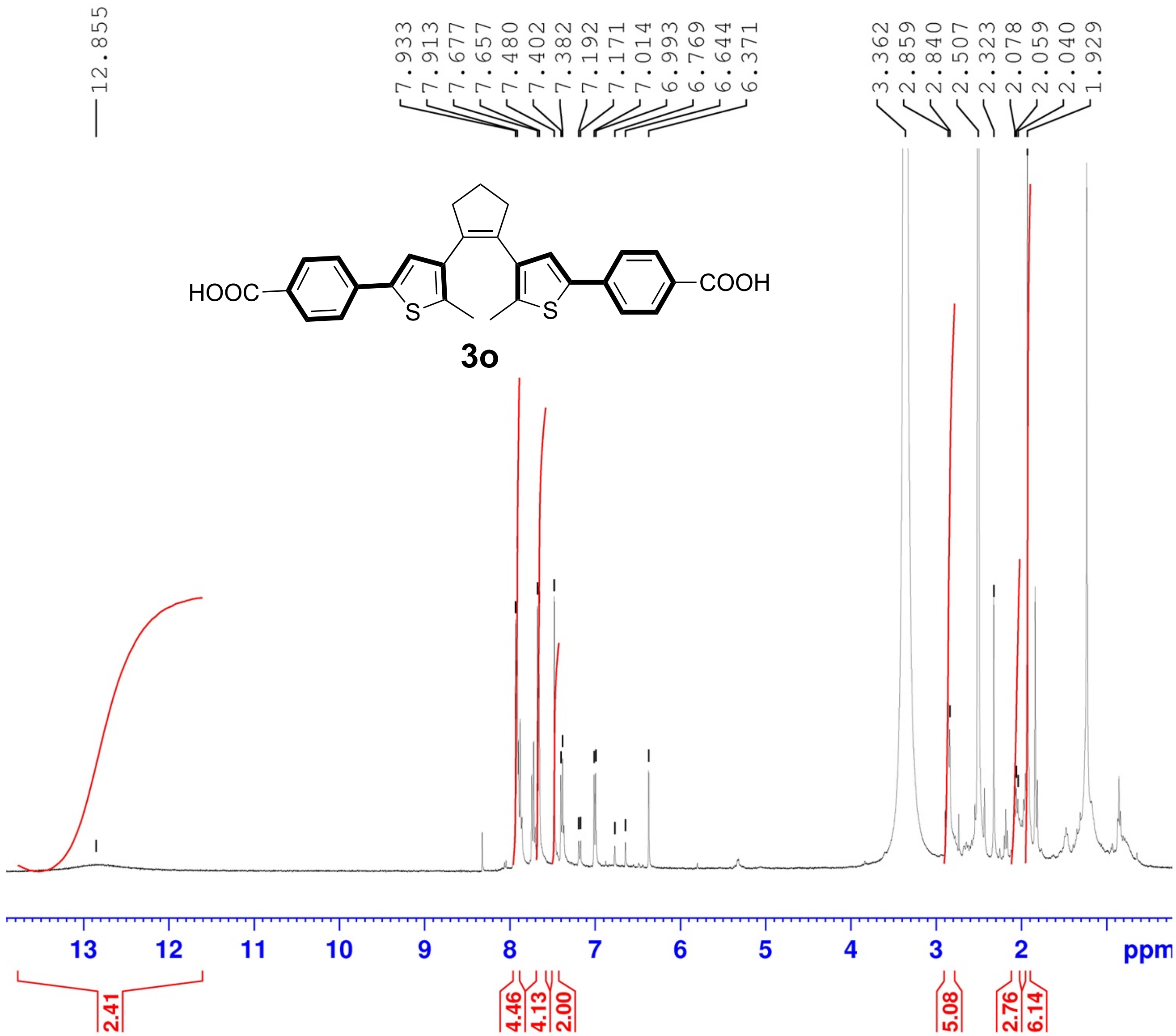

3 Reference

Park, J., Q. Jiang, D. Feng and H.-C. Zhou (2016). Controlled Generation of Singlet Oxygen in Living Cells with Tunable Ratios of the Photochromic Switch in Metal–Organic Frameworks. *Angew. Chem. Int. Ed.*, **55**(25): 7188-7193. doi: 10.1002/anie.201602417.
